# Combining 24-Hour Continuous Monitoring of Time-Locked Heart Rate, Physical Activity and Gait in Older Adults: Preliminary Findings

**DOI:** 10.3390/s25061945

**Published:** 2025-03-20

**Authors:** Eitan E. Asher, Eran Gazit, Nasim Montazeri, Elisa Mejía-Mejía, Rachel Godfrey, David A. Bennett, Veronique G. VanderHorst, Aron S. Buchman, Andrew S. P. Lim, Jeffrey M. Hausdorff

**Affiliations:** 1 Center for the Study of Movement, Cognition and Mobility, Neurological Institute, Tel Aviv Sourasky Medical Center, Tel Aviv 6423906, Israel; eitanash@tlvmc.gov.il (E.E.A.); erang@tlvmc.gov.il (E.G.); 2 Division of Neurology, Department of Medicine, Sunnybrook Health Sciences Centre, University of Toronto, Toronto, ON M4N 3M5, Canadaandrew.lim@utoronto.ca (A.S.P.L.); 3 Rush Alzheimer’s Disease Center, Rush University Medical Center, Chicago, IL 60612, USA; elisa_mejia@rush.edu (E.M.-M.); rachel_e_godfrey@rush.edu (R.G.); david_a_bennett@rush.edu (D.A.B.); aron_s_buchman@rush.edu (A.S.B.); 4 Department of Neurology, Division of Movement Disorders, Beth Israel Deaconess Medical Center, Harvard Medical School, Boston, MA 02215, USA; vvanderh@bidmc.harvard.edu; 5 Department of Physical Therapy, Faculty of Medical & Health Sciences and Sagol School of Neuroscience, Tel Aviv University, Tel Aviv 6997801, Israel

**Keywords:** heart rate, wearable sensors, hemodynamic response, autonomic function, aging, continuous monitoring

## Abstract

Hemodynamic homeostasis is essential for adapting the heart rate (HR) to postural and physiological changes during daily activities. Traditional HR monitoring, such as 24 hour (h) Holter monitoring, provides important information on homeostasis during daily living. However, this approach lacks concurrent activity recording, limiting insights into hemodynamic adaptation and our ability to interpret changes in HR. To address this, we utilized a novel wearable sensor system (ANNE*^@Sibel^*) to capture time-locked HR and daily activity (i.e., lying, sitting, standing, walking) data in 105 community-dwelling older adults. We developed custom tools to extract 24 h time-locked measurements and introduced a “heart rate response score” (HRRS), based on root Jensen–Shannon divergence, to quantify HR changes relative to activity. As expected, we found a progressive HR increase with more vigorous activities, though individual responses varied widely, highlighting heterogeneous HR adaptations. The HRRS (mean: 0.38 ± 0.14; min: −0.11; max: 0.74) summarized person-specific HR changes and was correlated with several clinical measures, including systolic blood pressure changes during postural transitions (r = 0.325, *p* = 0.003), orthostatic hypotension status, and calcium channel blocker medication use. These findings demonstrate the potential of unobtrusive sensors in remote phenotyping as a means of providing valuable physiological and behavioral data to enhance the quantitative description of aging phenotypes. This approach could enhance personalized medicine by informing targeted interventions based on hemodynamic adaptations during everyday activities.

## 1. Introduction

Hemodynamic function is dynamic and constantly adapting to maintain adequate blood flow and oxygen, which are critical for the varied behavioral repertoire of everyday living. In response to changing behaviors, motivational and environmental demands, HR, stroke volume, and vascular resistance are continuously modulated by baroreflexes and other inputs to maintain stable blood pressure (BP) and blood flow. Aging is accompanied by decreased baroreflex function, blunted compensatory responses to low BP, and increased BP variability. These changes may be exacerbated by medications, medical illnesses, or neurodegenerative diseases like Parkinson’s disease and other neurological disorders. Recent advances in remote monitoring technologies are revolutionizing our ability to assess cardiovascular function, with over 200 different devices now available for monitoring various cardiovascular parameters in community settings [1]. However, the assessment of hemodynamic adaptation during daily activities remains challenging [2]. Common behavioral transitions, e.g., sit-to-stand or stand–walk, occur hundreds of times a day and require intact hemodynamic adaptation. Thus, time-locked measures of behavioral transitions and HR are crucial for determining whether hemodynamic adaptation responses are adequate or are impaired. Recent studies have demonstrated that even light-intensity physical activity and simple postural changes can have significant impacts on cardiovascular health [3]. Impaired hemodynamic responses may manifest as a wide range of non-specific complaints and difficulties. These include transient symptoms like dizziness, orthostatic hypotension, brain fog, and blurred vision that can significantly impair function and quality of life [4]. In addition, older adults with impaired hemodynamic adaptation have an increased risk of falls, mobility disability, cognitive decline, and even dementia [5,6,7].

Prolonged recordings of HR or BP have been employed for many decades and have advanced the cardiac care of hypertension and arrythmias (e.g., Holter monitoring). These ambulatory recordings record HR changes during everyday living, supporting ecological relevance [8]. Yet, since these recordings do not record concurrent daily activities, prolonged HR or BP monitoring does not inform us of the potential causal behaviors of these changes or the adequacy of hemodynamic adaptation. Building on these traditional methods, advances in wearable technology have enabled the simultaneous monitoring of both heart rate and physical activity in free-living conditions, providing initial insights into the relationship between daily activities and cardiovascular responses [9]. While these studies have demonstrated the potential of combined HR–activity monitoring, and recent work has shown it has promising applications for cognitive assessment [10], the field still lacks a comprehensive, time-locked analysis of specific daily activities and their corresponding cardiovascular adaptations in large community-based samples.

To fill this knowledge gap, this study summarizes our initial efforts to deploy a novel wearable sensor system (ANNE*^@Sibel^*) that enables time-locked 24 h recordings of both HR and common daily living activities (i.e., lying, sitting, standing, walking) in more than 100 community-dwelling older adults. We describe the data collection, signal processing, and custom tools developed to extract 24 h metrics of HR and daily activity data from these recordings. We also describe the construction and initial results for a new “heart rate response score” (HRRS), based on the root Jensen–Shannon divergence, that summarizes the person-specific joint changes in HR during repeated occurrences of four different common daily activities.

## 2. Materials and Methods

### 2.1. Participants

Participants were recruited from the Rush Memory and Aging Project (MAP), a community-based cohort study of the chronic condition of aging, in which participants agree to an autopsy at their time of death. This study began in 1997 and biennial wrist actigraphy for up to 10 days was added in 2005. Participants who agreed to biennial actigraphy were eligible for the current study. The recruitment and deployment of the new ANNE sensor system began in April, 2023.

### 2.2. Time-Locked Collection of Heart Rate and Daily Activities

**Figure 1:**
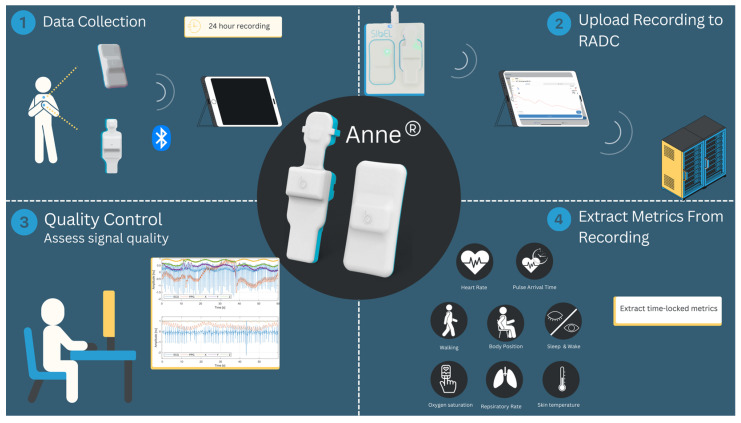
Illustration of the ANNE device. The system includes a chest-worn sensor that captures ECG and inertial measurement unit (IMU) data and a finger-worn sensor that records photoplethys- mography (PPG) and pulse oximetry data.

Continuous time-locked recordings of HR, accelerometer, gyroscope, and HR data were collected using the ANNE device, a novel sensor system consisting of two multi-sensor units (see Figure 1). A chest sensor, affixed to the participant’s chest, contains an inertial monitoring unit (IMU) with a triaxial accelerometer and triaxial gyroscope, from which physical activity data are derived, along with a thermometer and an ECG sensor. A second finger sensor, attached to the non-dominant hand, records photoplethysmography (PPG), pulse oximetry, and temperature. The two units are synchronized with millisecond precision.

A more detailed description of the ANNE device is provided in the Supplementary Materials.

### 2.3. Signal Processing and Data Extraction

In the present work, we focus on the ECG and IMU signals from the chest sensor.

#### Summarizing Beat-to-Beat Heart Rate

The ECG signals were processed using a wavelet transform to identify QRS complexes. Within each identified window, local maxima and minima were used to precisely locate R-peaks. The Hilbert transform [11] was applied to extract the envelope of each QRS complex, with the area under the curve used as a signal-to-noise ratio measure. Morphological features were extracted for each peak, and hierarchical density-based spatial clustering (HDBSCAN) [12] was used to group peaks into clusters. The peaks contained in clusters containing sequences of peaks with stable RR intervals within the physiological range of the human HR were identified as likely QRS complexes. The beat-to-beat HR was computed from these identified complexes and smoothed over a 5-beat rolling window to account for potential irregularities (e.g., atrial fibrillation). The mean HR obtained during the recording was determined for each subject. In addition, we calculated the standard deviation of the HR for each subject during the recording as a simple proxy of HR variability [13].

### 2.4. Identification of Four Daily Living Physical Activities

(1) *Pre-processing*: Signals were checked for wear time and sensor orientation.

(2) *Identifying Four Common Activities*: An algorithm automatically detected and segmented distinct periods of lying (supine), sitting, standing, and walking (further details are included in Supplementary Materials). Previously published analytical tools used to extract metrics from a lower back sensor that contained a 3D accelerometer and three gyroscope signals [7,14,15,16] were adapted to automatically identify different activities using the 3D accelerometer and three gyroscope signals obtained from the ANNE chest sensor.

To confirm that the pipeline that we adapted for the chest-worn sensor used in the present study successfully detected different activities, we compared its activity detection output with that obtained, simultaneously, via the previously validated lower back sensor pipeline. The comparison involved activities such as walking, lying, and transitions between upright and lying positions. The results, shown in Figure S1, show that the activity detection obtained using the chest sensor closely aligns with that obtained using the lower back sensor, demonstrating the suitability of the chest sensor for this application.

**Figure 2:**
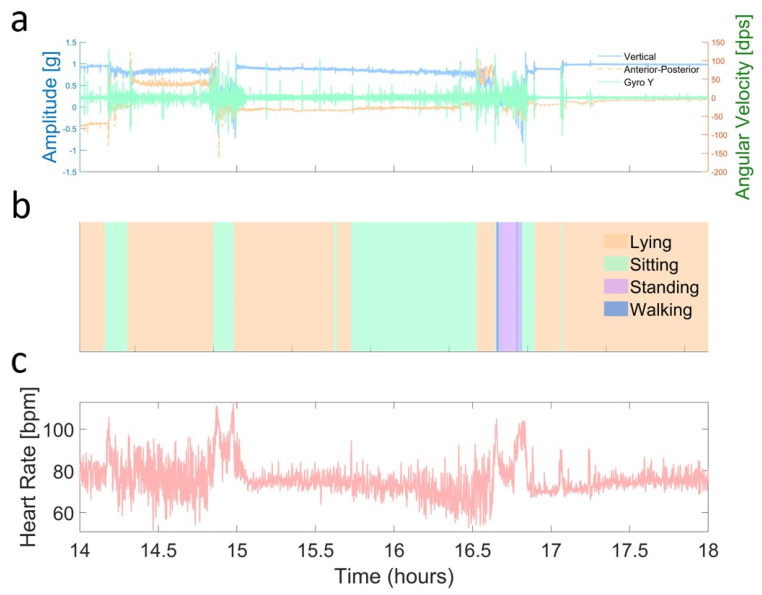
Defining body positions and activities over a 4 h window. (**a**) Example of accelerometer and gyroscope data, derived activity, and HR over a 4 h window. Accelerometer (vertical in blue, anterior–posterior in red) and gyroscope (green) data were smoothed with a 10-point moving average to highlight trends. (**b**) Barcode summary of different activities detected over the same 4 h period, with color-coded segments indicating lying (light orange), sitting (light green), standing (light purple), and walking (darker blue). The x-axis shows the actual time of day, rounded to the nearest half hour, for context. (**c**) Heart rate (light pastel red) measured during the same 4 h window, also smoothed with a 10-point moving average to illustrate fluctuations in beats per minute (bpm). Overall, this visualization demonstrates how raw sensor data (from the accelerometer, gyroscope, and ECG) correlates with inferred postures and activities, providing insights into the subject’s transitions and physiological responses.

(3) Identifying Transitions: A combination of the measures derived from the accelerometer and gyroscope data was used to determine transitions between different activities. Figure 2 illustrates how posture and activity are identified from the raw ANNE accelerometer and gyroscope signals. These signals provide distinct patterns for different body positions and activities, allowing for differentiation and accurate classification in quantifying the four activities, lying, sitting, standing, or walking, throughout the 24 h recording period. The time-locked recordings also allowed for the quantification of the HR during each of these four different activities. See Supplementary Materials for more information on the automated detection of each physical activity.

### 2.5. Analyzing Hemodynamic and Activity Measures Together

For each identified activity (i.e., lying, sitting, standing, and walking), we calculated the mean HR and its variance, which allowed us to characterizes the stable HR patterns seen during maintained activities. Based on the steady-state distributions of each of the four common activities, we defined an HR response score (HRRS) using the root Jensen–Shannon divergence (rJSD) between the HR distributions of consecutive activities.

The Jensen–Shannon divergence was selected over alternatives such as Kullback–Leibler divergence or Bhattacharyya distance due to its advantageous properties: it is symmetric (unlike KL divergence); always finite, even when the distributions have different supports; and bounded between 0 and 1, where 0 indicates identical distributions and 1 represents maximum dissimilarity. Its root form (rJSD) transforms the divergence into a proper metric, satisfying all distance axioms while maintaining these information-theoretic properties, making it particularly suitable for quantifying dissimilarities between physiological states.

Let *P* and *Q* be the probability distributions of the HRs for two consecutive postures. The root Jensen–Shannon divergence is defined as follows:

$$D_{rJSD}\left(P,Q\right)=\sqrt{\frac{1}{2}\sum_{k}P(k)\log\frac{P(k)}{Q(k)}+\frac{1}{2}\sum_{k}Q(k)\log\frac{Q(k)}{P(k)}}$$

The HRRS is then calculated as follows:

(1) $$HRRS=\sum_{\begin{array}{c} (i,j)\in T \end{array}}S_{ij}.D_{rJSD}\left(P_{i},P_{j}\right)$$

where *T* represents the ordered set of increasingly vigorous consecutive posture transitions: (lying, sitting), (sitting, standing), and (standing, walking). The sign function *s_ij_* captures the directional relationship between the HRs in consecutive postures:

(2) $$S^{ij}=\begin{array}{c} 1,\;if\;median(P_{j})\;\geq median\;(P_{i})\\ -1,\;if\;median(P_{j})<median(P_{i}) \end{array}$$

In simpler terms, the HRRS measures how much a person’s HR changes between different activities. It is expected that a higher positive score indicates that their HR increases as activities become more vigorous, suggesting a healthy, responsive cardiovascular system. Conversely, a lower or negative score may indicate reduced cardiovascular responsiveness. For example, consider a subject whose HR increases from lying to sitting and from sitting to standing, but decreases from standing to walking. In this example, the positive contributions from the first two transitions would be partially offset by the negative contribution from the standing-to-walking transition, resulting in a relatively low overall HRRS that reflects these varying patterns of cardiovascular responsiveness. While conventional statistical measures such as mean HR and standard deviation characterize overall heart function, the HRRS specifically quantifies changes in the HR distribution during different activities, potentially providing unique insights into how well the heart adapts to the specific demands of varied activities during daily living by accounting for both the magnitude and directionality of HR changes across different activities.

### 2.6. Clinical Measures

Demographic data and medical history, including diagnoses of heart conditions, were collected through self-reported questionnaires and chart reviews. Blood pressure (BP) measurements were obtained in both sitting and standing positions by a research assistant during the same home visit. The change in BP after transitioning from sitting to standing was used as a conventional proxy for cardiovascular responsiveness. Motor function was assessed using a composite score derived from 10 standardized tests evaluating manual dexterity, walking ability, balance, and strength [17]. Parkinsonian signs were evaluated using a 26-item modified version of the motor portion of the United Parkinson’s Disease Rating Scale (mUPDRS), which assessed bradykinesia, gait, rigidity, and tremor. Based on these assessments, participants were classified as having no parkinsonism (0 signs), possible parkinsonism (1 sign), or parkinsonism (≥2 signs) [18]. Their disability status was evaluated using standardized assessments, including the Activities of Daily Living (ADL) Index [19]. Late-life social activity was measured on a 1–5 scale across six types of social activities [20], with higher scores indicating more frequent social engagement. Physical activity was assessed using an adapted version of the 1985 National Health Interview Survey [21], which measures weekly hours spent carrying out five activities: walking for exercise, gardening, general exercise/calisthenics, bicycle riding, and swimming. For each activity, participants reported their engagement over the past two weeks in terms of the activity’s frequency and duration, which were then converted to hours per week.

### 2.7. Statistical Analyses

Mean and standard deviations were used to summarize group values. To analyze the relationships between the HRRS and clinical measures, we used different statistical approaches depending on the type of variable: Pearson’s correlation coefficient was used for continuous variables, while t-tests were employed for comparing subgroups defined by binary variables. All correlations were computed using Pearson’s correlation coefficient. Statistical significance was set at *p <* 0.05. All analyses were performed using MATLAB (version R2024b, The MathWorks Inc., Natick, MA, USA), with custom scripts used to handle the different types of variables. In this exploratory work, we did not adjust for multiple comparisons.

## 3. Results

### 3.1. Twenty-Four-Hour HR Measures and Clinical Correlations

Our cohort consisted of 105 older adults (mean age 83.5 ± 6.8 years; 31 males, 74 females). Their demographic, clinical, and cardiovascular characteristics are presented in Table 1. 

The overall mean HR across all subjects and postures was 76.04 ± 15.29 bpm, and the mean individual standard deviation of this HR was 11.9 ± 3.9 bpm. BP measurements showed mean values of 126.6 ± 17.2/73.6 ± 8.5 mmHg while sitting and 131.5 ± 19.2/79.3 ± 9.5 mmHg while standing. The mean change in BP when transitioning from sitting to standing was 5.1 ± 10.2 for systolic and 5.5 ± 7.3 mmHg for diastolic pressure. As might be expected, age was negatively correlated with the change in systolic BP after standing (r = −0.25, *p* = 0.021), while sex was not (t = 0.107, *p* = 0.915).

### 3.2. Twenty-Four-Hour Activity Patterns

**Figure 3:**
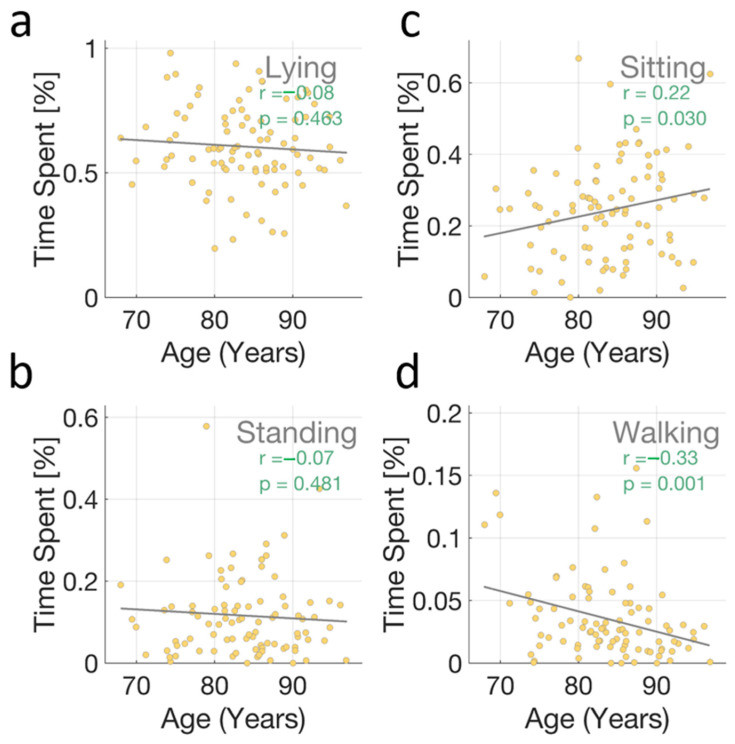
Age–activity correlations. Scatter plots showing the relationship between age and the relative time spent doing four different activities over a 24 h period: (**a**) lying, (**b**) sitting, (**c**) standing, and (**d**) walking. Each dot represents an individual participant. The dashed lines represent the fit of the linear regression. Pearson correlation coefficients (r) and corresponding *p*-values are displayed in each subplot. Notably, significant correlations were observed for sitting (positive correlation, r = 0.22, *p* = 0.030) and walking (negative correlation, r = −0.33, *p* = 0.001), indicating that older participants tend to spend more time sitting and less time walking. No significant correlations were found for lying (r = −0.08, *p* = 0.463) or standing (r = −0.07, *p* = 0.481).

Participants spent, on average, 48.43% of their time lying, 29.34% sitting, 16.14% standing, and 6.10% walking; see Figure S2. To understand how activity patterns relate to age, we examined correlations between time spent doing different activities and participant age (Figure 3). Interestingly, we found significant correlations for sitting and walking activities. Older participants tended to spend more time sitting (r = 0.22, *p* = 0.030) and less time walking (r = −0.33, *p* = 0.001). No significant correlations were observed for the time spent lying or standing (*p* > 0.4). None of these times were significantly correlated with sex (*p* > 0.5). The proportion of time spent standing was correlated with the change in systolic BP observed after transitioning from sitting to standing (r = 0.22, *p* = 0.045).

**Figure 4:**
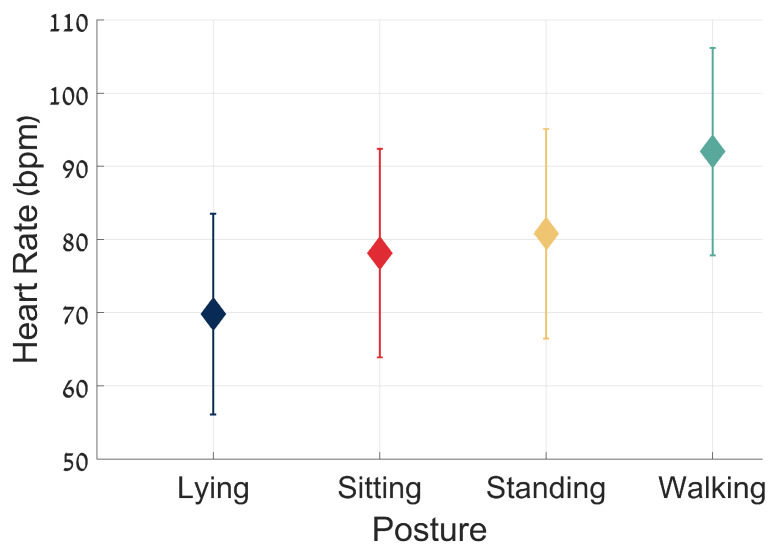
Average heart rate and standard deviation for the four main activities across all study subjects. The plot shows the mean HR (bpm) for each activity—lying, sitting, standing, and walking—with each represented by different markers and colors. Error bars indicate the standard deviation of the HR values for each activity. The data are aggregated from multiple subjects, with a minimum segment duration of 30 s per activity. The mean HR values are as follows: lying, 69.83 ± 13.72 bpm; sitting, 78.13 ± 14.25 bpm; standing: 80.79 ± 14.33 bpm; and walking, 92.04 ± 14.17 bpm. The Welch’s *t*-test *p*-values for consecutive activities are all smaller than 0.001, indicating significant differences between each pair of activities. The average HR increases, as expected, with the vigor of the activity, from lying to walking.

Figure 4 illustrates the average HR for the four main activities across all study subjects. As expected, the average HR generally increased from lying to walking activities, reflecting the walking activities’ increased physiological demands.

### 3.3. Combined HR and Activity Measurement

**Figure 5:**
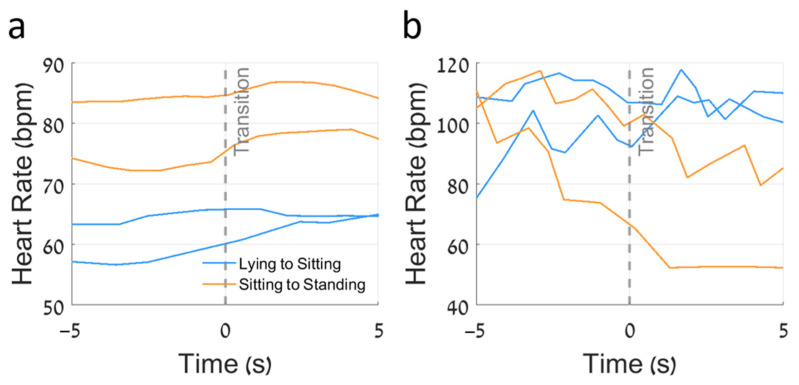
Heart rate responses to activity transitions in subjects with differing HRRSs. (**a**) Subject with a moderate-to-high HRRS of 0.58, demonstrating appropriate directional HR changes during transitions. The lying-to-sitting transitions (pastel blue) and sitting-to-standing transitions (pastel orange) show consistent increases in heart rate, as expected with more demanding postures. (**b**) Subject with a low HRRS of 0.22, exhibiting inconsistent directional HR changes during the same types of transitions. Despite transitions to more demanding postures, their HR sometimes fails to increase appropriately or even decreases, indicating less optimal cardiovascular adaptation. The gray dashed line at time zero represents the moment of transition. This figure illustrates how the HRRS captures the physiological appropriateness of directional HR changes, with higher HRRSs reflecting more consistent increases in HR with more demanding activities.

By synchronizing HR data with continuous activity measurements, we examined the relationship between HR and four different activities. Figure 5 illustrates how this combined approach reveals distinct patterns of HR adaptation to physical activity. Subjects with “robust” responses (Figure 5a) showed clear HR adjustments during an activity change, with noticeable increases in HR after moving from lying to sitting and from sitting to standing. In contrast, those with “weak” responses (Figure 5b) exhibited minimal HR changes, suggesting potential autonomic dysfunction and altered hemodynamic homeostasis. Note the stark contrast between the “robust” and “weak” HR responses to the lying-to-sitting or sitting-to-lying transitions. The HRRS quantifies and summarizes these adaptions during 24 h for each subject, as described in the next section.

### 3.4. Heart Rate Response Score

#### Quantifying HR Responsiveness in Daily Living

**Figure 6:**
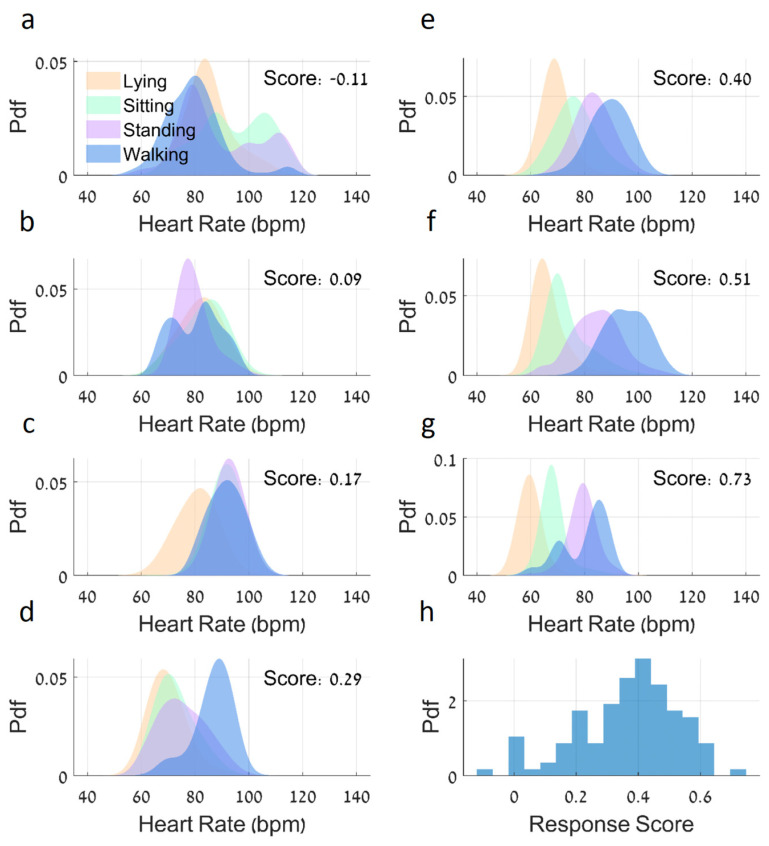
Heart rate distributions for different activities and accompanying HRRS distribution. (**a**) The upper left subplot shows the probability density functions (PDFs) for the HR of the subject with the lowest HRRS of −0.11, where their median HR for walking is lower than that for standing and sitting. (**b**–**g**) The next subplots display PDFs for the HR of subjects with progressively higher HRRSs, with increasingly separated distributions seen for different activities, with scores ranging from 0.09 to 0.73. Each subplot includes fitted distributions for HR values smoothed using a kernel density estimation with a bandwidth of 3 bpm. (**h**) Histogram of the HRRSs’ distribution for all subjects, which appears to follow a normal distribution. This figure demonstrates the variability in HR responses across different activities, with the HRRSs indicating the degree of separation between activity-specific HR distributions.

Figure 6 presents histograms of HR distributions for seven example subjects during different activities (panels a–g), along with the distribution of the HRRSs across all subjects (panel h). The subjects show a range of HRRSs, from −0.11 to 0.73. Subject A (Figure 6a) demonstrates an inverted HR response, with their median HR for walking lower than that for standing and sitting, resulting in a negative HRRS. In contrast, Subject G (Figure 6g) shows a clear separation of HR distributions across activities, with progressively higher median HRs for more vigorous activities, yielding a high positive HRRS.

The histogram of the HRRSs (Figure 6h) reveals an approximately normal distribution across the study population (with mean values of 0.38 ± 0.14), suggesting a spectrum of cardiovascular responsiveness to the different activities among these older adults.

### 3.5. Correlations Between HRRSs and Clinical Measures

#### 3.5.1. Heart-Related Measures

The conventional measure of cardiovascular responsiveness, the difference in systolic BP between sitting and standing positions, was associated with the HRRS values (r = 0.325, *p* = 0.003). In contrast, this difference in BP was not associated with the 24 h mean HR or 24 h SD of the HR (*p* > 0.22). Anecdotally, we note that in two participants who met the criteria for orthostatic hypotension, HRRS values tended to be lower (0.05, 0.22) compared to those who did not have OH (n = 103) (mean = 0.39 ± 0.14; range: −0.11 to 0.73; t = −2.596, *p* = 0.012). Participants taking calcium channel blocker medications (n = 17) displayed higher HRRS values (0.47 ± 0.10) compared to non-users (n = 79, mean = 0.37 ± 0.14; t = 2.362, *p* = 0.020).

#### 3.5.2. Correlations Between HRRSs and Activity-Specific HR Measures

As summarized in Table 2, the HRRS showed significant correlations with both overall and activity-specific HR measures. The strongest correlations were observed during sitting and standing activities, while the correlations during lying and walking were less pronounced.

## 4. Discussion and Conclusions

Leveraging new technology, the current study shows the feasibility of obtaining 24 h recordings of HR and simultaneous daily activities in large numbers of older adults during everyday living in their own homes and communities. These exciting and novel data fill an important gap in research on the prolonged monitoring of HR by linking these data to the varied activities that occur during these recordings. Time-locked recordings are critical to providing information on the spectrum of hemodynamic adaptations to postural changes caused by the hundreds of behavioral transitions occurring during everyday living. This report summarizes the development of a toolkit of algorithms needed for the post hoc extraction of multiple metrics from recordings of time-locked streams of behavioral and physiologic data. In addition to the novel 24 h heart rate and daily activity metrics, we also developed a measure linking both streams of data, called the “heart rate response score (HRRS)”. This metric summarizes the HR seen during four different activities over the course of 24 h recordings. A better HRRS was related to better cardiovascular function (e.g., changes in BP). Further studies are needed to further validate the HRRS using conventional clinic-based cuff BP measures, which are commonly used to identify orthostatic hypotension, an example of impaired hemodynamic adaptation when transitioning from sitting to standing. Further, studies on a larger number of older adults will be needed to determine the spectrum of the clinical consequences of impaired hemodynamic adaptation not only for sit-to-stand transitions, but also for the other activities quantified in this initial study. Our approach, using this new sensor system, is critical for providing information on the spectrum of impaired hemodynamic adaptations seen during daily living, which could not previously be studied due to the lack of time-locked recordings of HR and daily activities. A broader array of extraction tools will be needed to capture individual transitions between different activities. Moreover, further work will be necessary to determine the biology underlying hemodynamic adaptation. These data highlight the utility of deploying this system for the remote phenotyping of multiple behaviors together with physiologic data, which is crucial for advancing personalized medicine for targeted interventions that maintain independent living in aging adults.

Hemodynamic homeostatic responses maintain the delivery of blood, which is critical for all behaviors during daily living. In response to changing behaviors (e.g., posture or exercise) or motivational and environmental demands, the HR, stroke volume, and vascular resistance are continuously modulated by baroreflexes and other inputs to maintain stable BP [22]. Aging is accompanied by decreased baroreflex function, blunted compensatory responses to drops in BP, and increased BP variability; this may be amplified in Parkinson’s disease and other neurological disorders.

Clinically available approaches used to identify impaired hemodynamic adaptation include cuff-based sit-to-stand BP measurements in clinics and tilt-testing in specialized labs. Yet, these only provide a single snapshot in time. This is a limitation, as homeostatic hemodynamic responses are context-dependent and adapt differently to varied activities that are not captured by a single sit-to-stand measure in the clinic. Some adults appear normal in the clinic, but are dizzy getting out of bed at night. Some experience transient symptoms like brain fog, blurred vision, or unsteadiness that may impair their cognition and gait. Conventional ambulatory 24 h measures of BP and HR have advanced cardiac care. They do not, however, link the hemodynamic responses that are recorded to specific daily transitions, e.g., sleep-to-awake or sit-to-stand transitions, or let us know whether the HR response is appropriate. Recent advances in wearable sensor technology have demonstrated significant potential for the continuous monitoring of cardiovascular responses in free-living conditions, with studies showing strong correlations between consumer wearables and clinical-grade devices for measuring heart rate and activity patterns [2,23]. Analyzing different transitions that are time-locked with beat-to-beat hemodynamic adaptation responses is crucial to understanding the adequacy of hemodynamic adaptation. To address this need, we developed and implemented a comprehensive monitoring approach that combines these capabilities.

The current study focused on HR and physical activity during a 24 h recording. This continuous monitoring approach provides a quantitative view of cardiovascular responses to different activities during everyday living that is not possible with traditional, single-point measurements or conventional ambulatory HR monitoring. The chest sensor recorded ECG and tri-axial movement data, which enabled the precise synchronization of HR with physical activities. So, the metrics extracted from the recordings provide information on how HR changes before, during, and after different activities. Hence, this novel approach facilitates the 24 h remote digital phenotyping of hundreds of real-life transitions that could be thought of as multiple stress tests during the entire day, enabling the assessment of the adequacy of hemodynamic adaptation.

Our analysis focused on four main physical activities—lying, sitting, standing, and walking—which can be derived from the triaxial accelerometer contained in the chest sensor using extraction tools that have been described in prior publications (see the Supplementary Materials). These four common and frequent daily activities were chosen as they are performed by all older adults during daily living. As expected, the average HR increased when comparing lying to sitting, sitting to standing, and standing to walking, at the group level (Figure 4). At the same time, as depicted in Figure 6, at the individual level, these responses were heterogeneous. These findings could not be made without the time-locked recording of HR and physical activity. While our analysis focused on the most common daily activities in older adults, in the future this approach could be extended to other physical activities, such as stair climbing, stair descent [24], or stationary cycling [25], though these activities are less frequently performed by older adults [26].

By integrating HR patterns with these four different activities, we developed a quantitative metric of hemodynamic responsiveness that reflects real-world hemodynamic function in the community setting rather than in a lab environment. The HRRS showed associations with several clinical measures. Systolic BP differences between sitting and standing activities correlated with the HRRS values, and, anecdotally, participants with orthostatic hypotension (n = 2) displayed significantly lower HRRS values (mean = 0.14 ± 0.12) compared to those without OH (n = 103, mean = 0.39 ± 0.14; t = −2.596, *p* = 0.012). The overall 24 h mean HR was negatively correlated with HRRS, consistent with previous studies showing that a lower resting heart rate is associated with better cardiovascular health [27]. Activity-specific analyses revealed correlations between HRRSs and HR measures during different activities, with the strongest correlations observed during sitting and standing activities. These relationships align with previous research on cardiovascular adaptation during postural transitions [28,29].

Another important aspect of the current study is that it highlights the increasing importance of prolonged behavioral recordings. Remote prolonged phenotyping is becoming more feasible with the miniaturization of diverse body-worn sensors and the increased longevity of their batteries. Prolonged time-locked recordings have the potential to yield a wealth of new metrics that have previously not been available from individual signals, as well as from time-locked streams of data like HRRSs. HR variability (HRV) has been extensively studied and has significant diagnostic and prognostic value across various clinical conditions [30,31,32]. Similarly, recent work focusing on mobility has suggested that variability measures of multi-day mobility metrics may be a stronger predictor of outcomes compared to a single measures [33]. Further work will be needed to examine the associations between these data and HRV alone versus HRRS to determine if the integrated metrics provide more granular assessments of the critical facets of the complex aging phenotypes that are being recorded. The integrated metrics may lead to the identification of subtypes of older adults that may require targeted personalized therapeutic interventions.

The present study has strengths and several limitations. As mentioned above, to better understand the observed relationships, longitudinal studies would be informative. Our exploration of the associations between HRRSs and other measures was not hypothesis-driven, and we did not adjust for multiple comparisons. While our sample size exceeded 100 participants, individual responses showed much heterogeneity. So, it is important to confirm our findings in a larger and more diverse sample. The current study focused on only three streams of data recorded by the ANNE sensor. Further studies that incorporate additional cardiopulmonary metrics will likely fill in many current gaps in our understanding of the complexity of hemodynamic adaption in older adults. While 24 h summary data can advance our understanding, metrics that capture the individual transitions between different activities (Figure 5) will be needed to assess the adequacy of hemodynamic adaptation to everyday activities in older adults. Such an approach will be crucial for translating these measures to the clinical practice of treating aging adults. In addition, given the richness of the time-varying, time-locked physiological and physical activity signals that were recorded, additional analytic tools will be needed to maximize the utility of these signals. For example, multi-modal data fusion techniques, machine learning, and artificial intelligence for complex pattern recognition could be considered in order to address these challenges and opportunities.

This pilot study describes the feasibility of deploying unobtrusive sensors that can provide novel time-locked recordings of hemodynamic and daily living activities. These data highlight the potential for extending the utility of conventional 24 h HR metrics to include the remote phenotyping of the adequacy of hemodynamic adaptations to the postural and physiologic “stressors” that occur hundreds of times during the course of daily living. This approach has potential to offer investigators and ultimately clinicians a means to assess the efficacy of hemodynamic adaptation, which is not currently obtained during the ambulatory monitoring of community-dwelling older adults. These new hemodynamic metrics may prove to be informative on the biology of a host of clinical complaints in older adults that may indicate a reduced hemodynamic responsiveness that has been difficult to document during brief visits to clinics or tertiary care facilities but may be obtained via remote prolonged phenotyping.

In conclusion, current technology such as Holter monitoring has had a profound impact on cardiac care across the human lifespan, despite the lack of simultaneously recorded behaviors. The gaps in current technologies underscore the potential impact and contributions of this initial report of a sensor system that provides time-locked remote phenotyping of hemodynamic and daily living activities for a common cardiovascular disorder, orthostatic hypotension. The data presented demonstrate that our approach, which uses this novel sensor system, can fill in critical knowledge gaps about other important behavioral and physiological phenotypes. Remote phenotyping in the community setting, as people go about their normal routine, can assess a wide range of older adults, including those who are not usually studied because they cannot travel to participate in lab-based or tertiary health care center studies. Time-locked recordings could thus transform the remote phenotyping of aging adults, advancing our understanding of the complexity and orchestration of varied daily living behaviors and their link with critical physiological systems. The data obtained via remote phenotyping could provide novel targets for mechanistic studies, lead to innovative precision medicine treatments, and potentially result in a paradigm shift in the ongoing monitoring of drug studies and chronic health care, optimizing the health and well-being of our aging population.

## Figures and Tables

**Table 1:** Demographic statistics and heart-related variables of study subjects.

Variable	Value (Units)
**Demographic Variables**	
Age	83.5 ± 6.8 (years)
BMI	28.6 ± 6.7 (kg/m^2^)
Education	16.5 ± 3.05 (years)
Sex (Male/Female)	31/74
**Heart-Related Variables**	
Mean HR	76.0 ± 15.2 (beats per minute)
HR Standard Deviation	11.9 ± 3.9 (beats per minute)
Mean BP (Sitting)	127.4 ± 17.2/73.9 ± 8.5 (mmHg)
Mean BP (Standing)	131.2 ± 19.2/79.0 ± 9.5 (mmHg)
Orthostatic Hypotension	2 (1.9%)
History of Orthostatic Hypotension	24 (23.1%)
Cumulative Hypertension	64 (61.5%)
Cumulative Heart Disease	8 (7.7%)
Cumulative Claudication	12 (11.5%)
Cumulative Stroke	7 (6.7%)
Cumulative Congestive Heart Failure	10 (9.6%)

**Note:** All historical variables are based on self-reported data. Cumulative variables indicate reported conditions in a subject’s history or in at least one follow-up cycle up to and including the current cycle. Claudication refers to reported pain in calves while walking. Hypertension, heart disease, stroke, and congestive heart failure are based on self-reported diagnoses by a health care professional.

**Table 2:** Correlations between HRRSs and activity-specific HR measures.

Metric	Correlation (r)	*p*-Value	Significance
*24 h Measures*			
HR Mean	−0.26	0.002	**
HR Standard Deviation	−0.09	0.372	ns
*Activity-Specific Mean HR*			
Lying	0.13	0.192	ns
Sitting	−0.39	<0.001	***
Standing	−0.29	0.002	**
Walking	−0.16	0.110	ns
*Activity-Specific HR Variability*			
Lying SD	−0.20	0.047	*
Sitting SD	−0.27	0.004	**
Standing SD	−0.26	0.006	**
Walking SD	−0.23	0.016	*

*** *p* < 0.001, ** *p* < 0.01, * *p* < 0.05, ns: not significant.

## Data Availability

All data included in these analyses are available via the Rush Alzheimer’s Disease Center Research Resource Sharing Hub, which can be found at www.radc.rush.edu (accessed on 17 April 2023). It has descriptions of the studies and available data. Any qualified investigator can create an account and submit requests for deidentified data.

## Supplementary Materials

The following supporting information can be downloaded at: https://www.mdpi.com/article/10.3390/s25061945/s1, Figure S1: comparing the detection of body position based on a lower back sensor to a chest sensor. The body position vector is assigned the following values: 0 = upright (stand/sit), 1 = walking, 1.5 = upright to lying, 1.8 = lying to upright, 2 = lying. The figure shows two closely matching lines representing activity detection by the lower back sensor and the chest sensor, demonstrating a close alignment between the two methods of detecting posture across various activities. The table below compares the start time of activity detection by the lower back sensor and the chest sensor, with errors and standard deviations (STD) provided in seconds. Figure S2: comparison of average time spent in each activity across all subjects. This figure illustrates the average time spent in four different activities—lying, sitting, standing, and walking. The bars represent the mean time (in percentages) spent in each activity. Table S1: comparison of start time detection by lower back sensor vs. chest sensor.
