# Supplementary material for: Combining 24-Hour Continuous Monitoring of Time-Locked Heart Rate, Physical Activity and Gait in Older Adults: Preliminary Findings
Source: Sensors (Basel). 2025 Mar 20;25(6):1945. doi: 10.3390/s25061945 (PMC11946096; doi:10.3390/s25061945)
Supplement: Supplementary file 1 [file sensors-25-01945-s001.zip › sensors-3462966-supplementary.pdf]

Supplementary Materials

Section S1 Assessment with the ANNE® System

Device: The Anne® system is an integrated system of synced wearable device created by Sibel Health, Inc (Chicago, IL, USA). This system is comprised of two independent components (Figure 1). The Anne® Chest Sensor includes two electrodes for measuring the electrocardiogram (ECG); a sensor to measure skin temperature; and a tri-axial accelerometer and tri-axial gyroscope to assess activity and movement. The Anne® Limb Sensor measures skin temperature and transmittance photoplethysmography (PPG), an optically derived signal which varies with blood volume changes. The PPG sensor features red and infrared wavelengths, which allow for the estimation of oxygen saturation. The battery life of both sensors is more than 36 hours. Moreover, they are internally synchronized, which implies that time-based metrics that require signals to be acquired precisely at the same instant can be reliably extracted from this system. In addition to the raw signals, the Anne® system provides a vital signs file which includes measures of temperature and oxygen saturation, performed every second. Subjects consented to wearing the ANNE system during an overnight recording.

Sensor Placement. Placement was during the day and the recordings continued until the following morning to obtain recordings lasting 18-24hrs. Before starting the recording, a research assistant synchronized both fully charged sensors. The chest sensor was fixed to the chest of each subject using a special adhesive that also features conductive gel to improve ECG signal quality. The chest sensor was placed just below the suprasternal notch, with the top of the sensor roughly aligned with the inferior border of the clavicles. The limb sensor was placed on the index finger of the non-dominant hand of each subject. An adhesive was wrapped around the sensor to keep the it in place during the recording and to reduce movement artifact from the PPG signal. Sensors were affixed during the day. Subjects were instructed to take the sensors off after they woke up the next day. Following placement, the RA started the recording using a mobile app provided by the manufacturer. The recorded data was then downloaded to a secured server for post-hoc processing and data extraction.

Signal preprocessing. The initial step for processing the data was to determine the recording termination time. This was done using the temperature information collected from both locations. Three potential termination times were considered based on the temperature measures. Firstly, the locations at which the temperature measured from the chest was higher than a threshold based on the final temperature measured was considered as a potential termination time. The second potential termination time was determined as the duration of the recording from the chest sensor after removing portions of the signal that had repeated temperature measures for at least 100 seconds. And finally, the third potential termination time was set as the time at which (a) the difference between chest and limb temperature measures was lower than a threshold; (b) the temperature measured at the chest was greater than a threshold based on the final temperature measured from this location; and (c) these conditions were met for a sufficiently long period of time. The final termination time was chosen as the minimum between these three potential times. The recordings were then segmented between the last time at which both sensors started recording data and the identified termination time.

Electrocardiography. The ECG signals are sampled by the Anne® at around 512 Hz. However, some data could be lost due to firmware issues. A linear interpolation was performed to convert this into a regularly sampled time series. QRS complexes were detected using an approach based on Wavelet filtering, and the quality of the R-to-R interval was assessed using a HDBSCAN clustering approach. This clustering algorithm uses features from the QRS complexes identified to determine the quality of the QRS complex. Then, using the R-to-R intervals of good quality, the

instantaneous HR was estimated. Finally, the resulting ECG signal was down sampled to 100 Hz for further processing, and a quality metric based on regularity of instantaneous HR was obtained.

Accelerometer. The data obtained from the triaxial accelerometer was resampled to 100 Hz to match the sampling rate of other devices used in similar studies. Each channel from the accelerometer was filtered using a 5th order, lowpass Butterworth filter with a cutoff frequency of 10 Hz, followed by a moving average filter with a window duration of 100 milliseconds. Body position was determined based on the accelerometer data using the following algorithm: Lying position was identified by examining the mean value of the vertical axis in 1-second windows. If the mean was lower than 0.55 (corresponding to an angle between 0 and 33.36 degrees from the ground), the posture was marked as lying. To ensure stability of the measurement, a lying posture was only confirmed if detected continuously for at least 5 minutes. After identifying a lying posture, the specific lying position was determined using the anteroposterior (AP) and mediolateral (ML) axes:

$$\text{Lying Position} = \begin{matrix} \text{Back}, & \text{if Deg} \geq 135^\circ \\ \text{Right side}, & \text{if } 45^\circ<\text{Deg}<135^\circ \\ \text{Lefl side}, & \text{if}-135^\circ\leq-45^\circ \\ \text{Lefl side}, & \text{if}-135^\circ\leq-45^\circ \end{matrix} \qquad (1)$$

where Deg is the angle calculated using the AP and ML axes. Non-lying postures (sitting, standing, walking) were classified based on the intensity and pattern of movement in the vertical axis.

Additionally, we calculated the Euclidean Norm Minus One (ENMO) to quantify overall movement intensity:

$$ENMO = \sqrt{x^2 + y^2 + z^2 - 1} \qquad (2)$$

where x, y, and z are the accelerations in the three orthogonal axes. It's worth noting that for laboratory protocol validations, a slightly different approach was used: the minimum 5-minute rule for lying detection was not applied, and the vertical axis threshold was set to 0.5 (corresponding to angles of 0-30 degrees to declare the activity as lying).

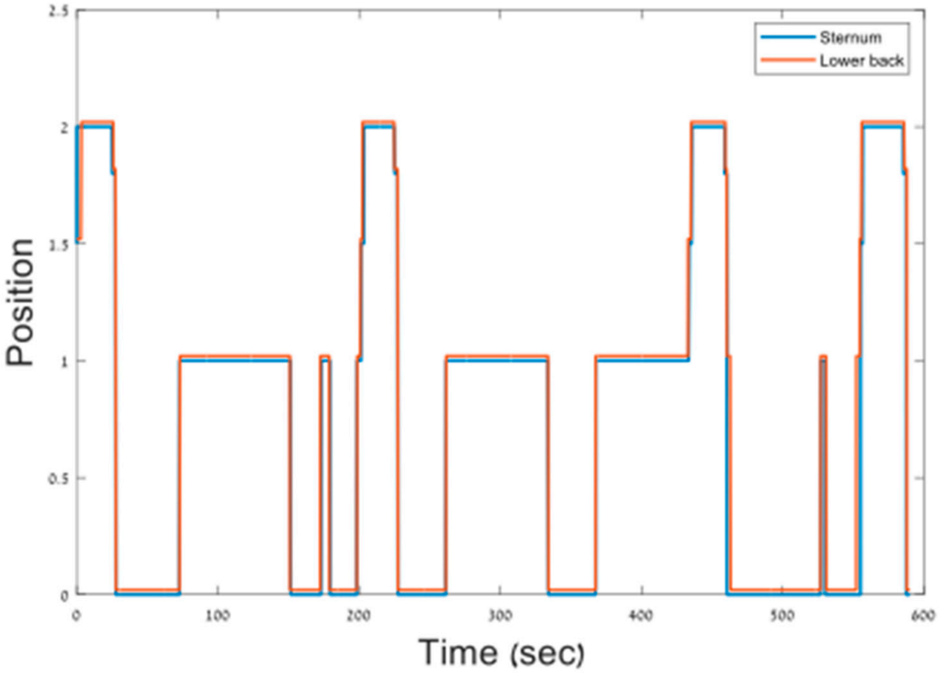

Figure S1. Comparing the detection of body position based on a lower back sensor to a chest sensor. The body position vector is assigned the following values: 0 = upright (stand/sit), 1 = walking, 1.5 = upright to lying, 1.8 = lying to upright, 2 = lying. The figure shows two closely matching lines representing activity detection by the lower back sensor and the chest sensor, demonstrating a close alignment between the two methods of detecting posture across various activities. The table below compares the start time of activity detection by the lower back sensor and the chest sensor, with errors and standard deviations (STD) provided in seconds.

Table S1. Comparison of start time detection by lower back sensor vs. chest sensor.

Activity	Error [s]	STD [s]
Walking	0.376	0.323
Lying	0.312	0.984
Upright to lying	-0.03	0.336
Lying to upright	0.787	1.26

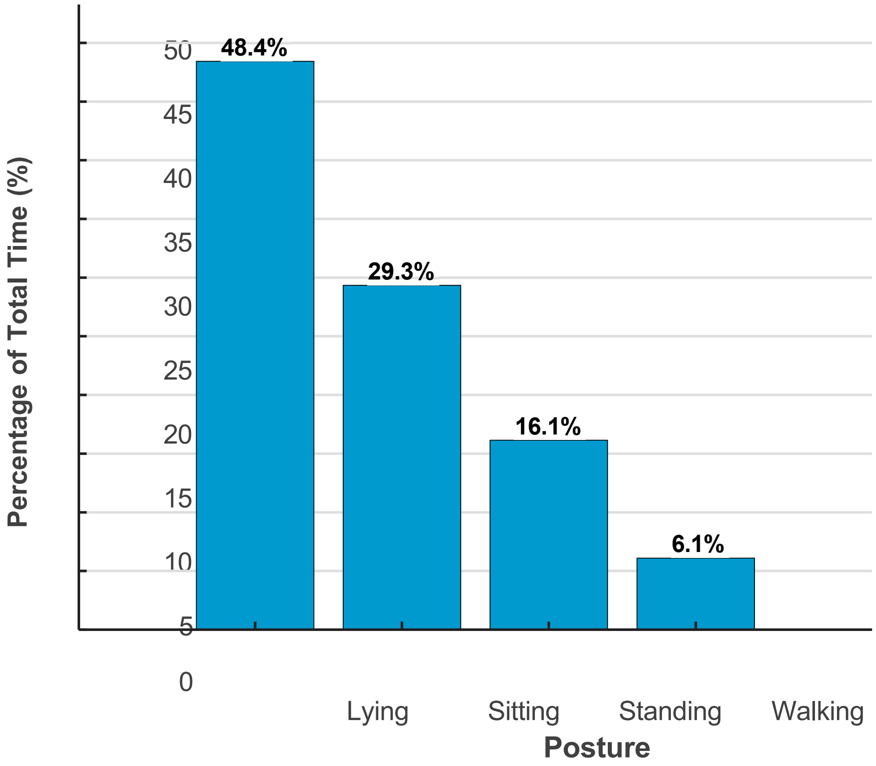

Figure S2. Comparison of Average Time Spent in Each activity Across All Subjects. This figure illustrates the average time spent in four different activities—Lying, Sitting, Standing, and Walking. The bars represent the mean time (in percentages) spent in each activity.
